# Optimization of AUTOSAR Communication Stack in the Context of Advanced Driver Assistance Systems †

**DOI:** 10.3390/s21134561

**Published:** 2021-07-02

**Authors:** Răzvan Bogdan, Mihaela Crișan-Vida, Darius Barmayoun, Loredana Lavinia Staicu, Robert Valentin Puiu, Mădălina Lup, Marius Marcu

**Affiliations:** 1 Faculty of Automation and Computers, University Politehnica Timisoara, 300223 Timișoara, Romania; mihaela.vida@upt.ro (M.C.-V.); LoredanaLavinia.Staicu@hella.com (L.L.S.); RobertValetin.Puiu@hella.com (R.V.P.); madalina.lup@hella.com (M.L.); marius.marcu@cs.upt.ro (M.M.); 2 Research Center for Engineering and Management, University Politehnica Timisoara, 300223 Timișoara, Romania; darius.barmayoun@student.upt.ro; 3 Vitesco Technologies Engineering Romania, 307285 Timișoara, Romania; 4 Hella Company, 307200 Timisoara, Romania

**Keywords:** AUTOSAR optimization, radar sensor, remote testing, radar testing, memory usage, runtime measurements, ADAS

## Abstract

New trends in the automotive industry such as autonomous driving and Car2X require a large amount of data to be exchanged between different devices. Radar sensors are key components in developing vehicles of the future, therefore these devices are used in a large spectrum of applications, where data traffic is of paramount importance. As a result, communication traffic volumes have become more complex, leading to the research of optimization approaches to be applied at the AUTOSAR level. Our paper offers such an optimization solution at the AUTOSAR communication level. The radar sensor is accessed in a remote manner, and the experiments aimed at performance measurements revealed that our solution is superior to the Full AUTOSAR implementation in terms of memory usage and runtime measurements.

## 1. Introduction

Technological developments of the automotive industry are currently increasing at an accelerated pace. New trends such as autonomous driving [1,2] and Car2X [3,4] require a detailed understanding of the interaction environment. Therefore, vehicles are becoming more and more interconnected between each other, as well as with the surrounding environment. Their capabilities are enhanced by intelligent software modules, contributing to an increase of a vehicle’s features. Specialized sensors are needed in the case of autonomous driving in order to enable efficient 360° environment recognition for the entire vehicle. Given the complexity of the operational conditions, Advanced Driver Assistance Systems (ADAS) have emerged to reduce life-threatening situations that might arise during the functioning time [5].

The Advanced Driver Assistance System is a vehicle-based intelligent safety system that offers support to drivers in terms of road and traffic safety. This module is monitoring, warning, and even controlling the vehicle with partial and complete override of the driver’s actions. Our initial data in this regard were presented in a conference paper [6]. Although ADASs are available in the most diverse forms, they all share the same purpose: making the driving experience safer and more comfortable [7]. The ADAS market is constructed taking into consideration the component types and the regions they are to be used in. The first category includes system and sensor types. The system type segment is subdivided into blind spot object detection systems, tire pressure monitoring systems, drowsiness monitoring systems, adaptive cruise control systems, adaptive front-lighting systems, intelligent parking assist systems, night vision systems, lane departure warning systems, and driver monitoring systems. The sensor type category includes radar, image sensor, ultrasonic sensor, LiDAR sensor, infrared sensor, and laser. It has been proven [8,9,10] that the implementation of advanced driver assistance system applications, such as lane departure warning systems and adaptive cruise control, night vision, or blind spot detection systems, has led to a significant reduction in the number of car accidents. According to the policy of the New Car Assessment Program (NCAP), modern vehicles must demonstrate the achievement of specific safety ratings in order to encourage significant safety improvements in state-of-the-art car designs. Additional support in this direction comes from governmental agencies focused on road safety. All these industrial, social, and public directions are expected to generate an increase in the advanced driver assistance system market in the future, requiring optimized communication implementations [11].

Modern sensors such as ultrasound and laser sensors (LiDAR sensor) and, of course, surround-view cameras ensure safe distance recognition and, in addition, recognition of the driving environment. A (central) control unit processes the data and converts it into signals, visual messages, or even active reactions. Today these actions usually occur digitally and within a fraction of a second. Due to the variety of systems available and all the individual solutions offered by different manufacturers, it is impossible to make a general statement as to which sensor system or sensor generation would be suitable for use in any particular application. Vehicle manufacturers use the most diverse driver assistance systems, practical combinations, and new technologies in all different vehicle classes [12].

Traditionally, electronic control units (ECUs) are developed in a hardware-dependent paradigm, taking into consideration the specific architectural components of the hardware setup. This approach requires new software to be created for each and every hardware upgrade. Over the years, the automotive industry has faced slow-downs in the development cycles of new products due to hardware limitations. Moreover, the reliability and reusability of software was reduced. As a result, several German automotive original equipment manufacturers (OEMs) started the Open Systems and Electronics in Motor Vehicles (OSEK) and the Automotive Open System Architecture (AUTOSAR) initiatives, in order to develop “an industry standard for an open-ended architecture for distributed electronic control units in automobiles” [5]. AUTOSAR aims to provide standard interfaces by offering hardware-independent application software that can be distributed in various components of the vehicle’s ECUs through the runtime environment layer. 

With a rapidly growing automotive industry moving towards intelligent systems and autonomous driving, the AUTOSAR platform is one of the solutions to overcome scalability and interoperability challenges. Autonomous driving requires a large number of sensors, thus increasing the amount of network participants and critical data to be processed. This is the reason why communication traffic volumes have become more complex and optimization approaches are researched to be applied at the AUTOSAR level [3,5]. Our work investigates the optimizing possibility of large data transmission at the AUTOSAR level. According to the literature review we have conducted, this particular approach has not yet been addressed in detail in the scientific literature. In a previous work [6], we started to explore the design and implementation of a similar solution. However, the system presented in [6] showed some intermediary results aimed at the radar sensor verifications. Therefore, the findings and results of the current proposal are focused on providing new scientific insights for further projects of similar characteristics, by describing the used methodologies, technical hardware and software implementations, testing procedures, lessons learned, and limitations.

Facing a global crisis of the Covid-19 outbreak, the automotive industry is introducing new testing procedures for embedded system applications, such as remote testing [13]. In this case, specific equipment are forming complex testing laboratories that should be accessed from any distance and at any given time. Our research is based on the motivation to offer such solutions when dealing with radar sensors. This is the reason for integrating remote testing capabilities with the radar sensor environment. 

Based on the observations presented above, the research questions of this paper are formulated as follows: (RQ1) To build the solution needed for the AUTOSAR communication optimization; (RQ2) To build the remote testing architecture and environment that is needed in order to access and test the optimization at AUTOSAR level; (RQ3) To analyze our system in terms of performance and runtime measurements, in order to offer clear scientific results regarding our solution that will benefit researchers in future work with similar characteristics.

The rest of the paper continues with: Section 2 presenting the fundamentals on AUTOSAR, followed by Section 3 containing the literature review on AUTOSAR and radar sensor optimization as well as AUTOSAR testing. Next, the work presents the system architecture and the three main features: AUTOSAR optimization solution, accessing the radar sensor in a remote manner, and the implementation of the performance tests module. These are all presented in Section 4. The following part of the paper, Section 5, presents the experiments we have conducted and the obtained results, while Section 6 is reserved for the conclusions of our research.

## 2. AUTOSAR Basic Concepts

Automotive Open System Architecture (AUTOSAR) was founded in 2003 and is a worldwide development partnership of automotive manufacturers. The goal of this consortium is to create and establish an open, robust, and standardized software architecture for automotive ECUs. This design includes scalability to a spectrum of vehicle and platform variants and portability of software, everything being based on the attributes of availability, reliability, and safety requirements. It is also aimed at maintaining the development process based on a predefined “Product Life Cycle” [1].

**Figure 1:**
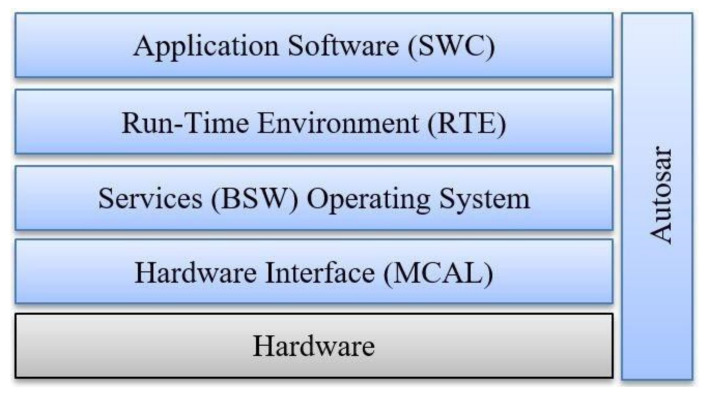
AUTOSAR Architecture.

**Figure 2:**
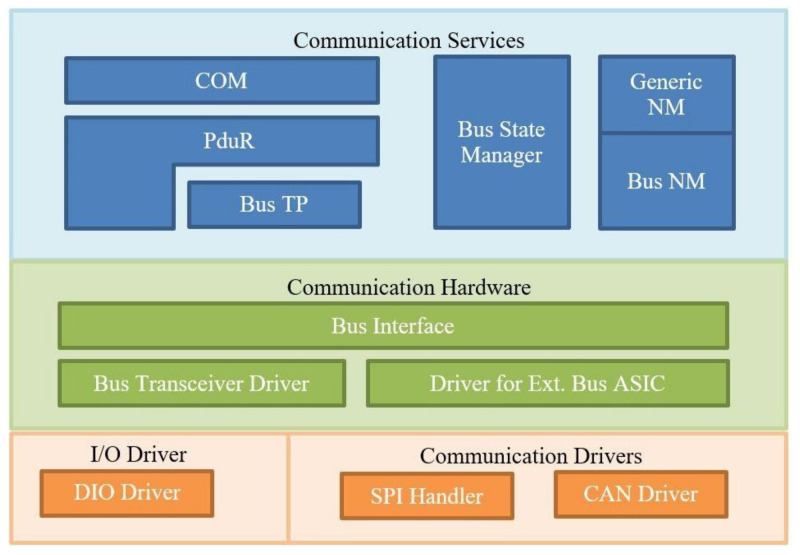
Bus Communication Stack.

The AUTOSAR development process (Figure 1) is implemented across the following software layers: MCAL (Microcontroller Abstraction Layer), Basic Software Layer (BSW), Run-Time Environment (RTE), and Application Layer. The Runtime Environment (RTE) has the role of decoupling the network from the Software Components (SWCs) of the application and providing uniform services in the form of Basic Software (BSW), such as system and diagnostic services, bus communication, IO access, and memory management. The BSW Communication Stack consists of the Services Layer, ECU Abstraction Layer, and the Microcontroller Abstraction Layer (MCAL). The AUTOSAR Communication Stack (Figure 2) offers the communication services for the BSW module, as well as for the Application Layer.

The Communication (COM) module is situated between the RTE and the PDU router, being responsible for providing access to the signals needed in the Application Layer. It also provides PDU level access to lower layers independent of the used protocol. At the transmitter, it will pack all the signals into a PDU, while at the application will unpack the received PDU in order to offer signal level access for the application.

The Protocol Data Unit Router (PduR) module is part of the Services Layer and will route the PDU to the specific bus interface module. It offers services for the routing of PDUs between: Transport Protocol modules; Communication Interface modules; Diagnostic Communication Manager (DCM) and Transport Protocol modules; COM and communication interface modules or I-PDU Multiplexer; IPDU-Multiplexer; and communication interface modules.

The basic services accessible through the Bus Transport Protocol (TP) module are: segmentation of those messages that have a payload of more than 8 bytes and transmission of the messages with flow control and reassembling the segmented messages at the receiver. The Bus State Manager (SM) module will implement the control flow for the respective bus. The purpose of the Bus Network Manager (NM) module is to manage the transition between normal operation and bus-sleep mode of the network. The Bus Interface module is part of the ECU abstraction layer and functions as an interface between the hardware abstraction layer (at the lowest level) and the service layer (above the Bus Interface module). External Bus Driver provides bus specific transceiver access to the upper layer services [1,5].

With various complex modular embedded software components within modern ECUs, different automotive safety integrity levels (ASIL) must be reached during the implementation of software functions. ASIL is defined by the ISO 26262—Road vehicles—Functional safety standard as an attribute of a software function implemented, for example, in an embedded system. As a result of a threat analysis and risk assessment of the product under development, the ASIL is derived indicating the quality level at which the software function must be implemented [14]. The ECU software can consist of both safety-relevant and non-safety-relevant components; therefore, different ASIL levels must be taken into consideration. In this case, the ISO 26262 standard states that either the highest ASIL rating-related methods must be followed during the development phase, or software components with lower ASIL rating must have interface freedom from the components with higher ASIL rating [15].

To ensure the required interface freedom between the various software components, AUTOSAR functional safety mechanisms have been implemented to prevent, detect, and mitigate possible software and hardware faults. Fault groups such as memory, timing, exchange of information, and execution are defined by the ISO 26262 standard. As an example, by storing OS applications in different memory regions, the AUTOSAR OS allows interfacing freedom for memory faults [14]. Therefore, memory partitioning protects the OS applications from one other during code execution. Besides the functional safety mechanisms, various other functional safety measures of AUTOSAR support the development of safety-relevant software.

In the same quality-related context, Automotive Software Process Improvement and Capability Determination (ASPICE) aims to evaluate the software development process performance of an organization and highlights software development base practices. Namely, defined as a framework to be applied by automotive OEMs, ASPICE is used to determine and evaluate the process of software development in the automotive industry. Without considering the safety aspect, ASPICE allows determining the capacity of an organization of delivering software products. However, to ensure safety-relevant requirements, an organization must prove compliance to both ASPICE and ISO 26262. Covering the whole development process, ASPICE provides the needed framework for the implementation of the functional safety standard, ISO 26262. For a better understanding of the connection between the two, ISO 26262 can be regarded as an extension of the ASPICE defined software development aspects from a functional safety perspective. Even if the ASPICE and ISO 26262 standards are different in many aspects, similarities can still be found in areas such as traceability of work products and change management.

## 3. Previous Work

This section presents the state-of-the-art literature regarding the main topics of our paper, AUTOSAR communication and optimization, as well as AUTOSAR testing.

### 3.1. AUTOSAR Communication

In their work, Anna Arestova et al. [3] address the communication challenge and propose an integration approach of the AUTOSAR Adaptive Platform, a machine-to-machine communication technology named Open Platform Communications Unified Architecture (OPC UA) and Time-Sensitive Networking (TN). Just by combining the use of TSN and OPC UA, the communication overhead is drastically reduced, enabling a flexible communication between various devices.

However, the communication optimization is reliable only if the data flow is secure. Cinzia Bernardeschi et al. [16] propose a method to check the data secure flow in security annotated AUTOSAR models. This method is based on an analysis of the information flow and an abstract interpretation. Their work aims to improve security in automotive communications, as recent studies have concluded that intruders can compromise the operability of modern automotive electronics systems. AUTOSAR modelling extensions that address cybersecurity relevant requirements have been defined while a code generation tool that synthesizes the appropriate services to use was developed. These automatically generated security components have proven to drastically reduce common errors during the development phase. Following the same cybersecurity path, Pietro Biondi et al. [17] present the proof-of-concept of the TOUCAN protocol. This security protocol follows the AUTOSAR standard and is designed to secure Controller Area Network communications. Thus, the goal of this protocol is to establish secure communications of CAN frames. The authors concluded that TOUCAN adds the needed security to the CAN communications while negligibly affecting the runtime. Testing the protocol outside a professional automotive environment has further proven the usability ease of the TOUCAN protocol. Another study conducted by Haichun Zhang et al. [18] underlines the CAN bus protocol vulnerabilities. An in-vehicle CAN security evaluation tool, CANsec, that simulates malicious attacks is proposed in order to evaluate the security risks. Moreover, the usage of CANsec was demonstrated by the authors even when no information from the vehicle manufacturer was provided.

The high demand of computational power of the electronic control units of the automotive industry lead to a new solution for replacing the widely used single core processors. Having a higher computational power and energy efficiency, the use of multi core electronic control units has rapidly increased. As a result, numerous works propose optimization methods for their use based on the AUTOSAR standard. Aiming to minimize the communication cost, Priyanshi Gupta et al. [19] propose an algorithm for an efficient mapping of AUTOSAR runnables in multicore automotive embedded systems. The solution aims to solve the communication overhead created by the data dependencies of the periodic AUTOSAR runnables and tasks, trying to map runnables from single core to multi core. The algorithm is presented as a possible solution for the homogeneous multicore systems. Addressing the optimization of runnable-to-task mapping of the multi core electronic control units, Thomas Wilhelm and Raphael Weber [20] propose a solution of automating two process steps, where the initial steps of runnable-to-task mapping and the optimization in the AUTOSAR platform are optimized. The initial configuration that balances the core utilization is automated by using constraint programming while an evolutionary algorithm is used by the authors to optimize an already existing configuration. It is also shown in this paper how the AUTOSAR implicit design constraints influence the modeling of the evolutionary algorithm. Aiming to reduce busy waiting in AUTOSAR, Robert Hottger et al. [21] propose a concept of Task-Release-Delta-based Runnable Reordering (TDRR). In order to achieve reduced task response times, increased parallel efficiency, and improved timing predictability, some AUTOSAR runnables are reordered. Their work was successfully applied to AMALTHEA models by using TDRR to execute tasks with different off-line calculated runnable orders. Furthermore, the experiments with industrial use cases have proven the ability of the solution to improve system performance by reducing task response times. Going a step further, Sebastian Kehr et al. [22] present a latency-aware parallelization technique, Parcus, that combines runnable and task level parallelism. This technique is used for a robust and energy-aware parallelization of the AUTOSAR legacy applications. Moreover, an evolutionary algorithm is presented in order to explore the large number of scheduling possibilities. Considering the latency and processor frequency, a parallel schedule quality metric quantifies the success of the parallelization. The applicability of the Parcus technique was demonstrated by the authors with a real diesel engine management system.

Optimization opportunities can arise in any direction, and this is proven by Qingling Zhao et al. [23] as their paper optimizes the design of AUTOSAR models with mixed-criticality scheduling and preemption thresholds. As a result of the development of a schedulability analysis of mixed-critically embedded systems, two algorithms, PA-DMMPT and HeuPADMMPT, are proposed. These algorithms aim to assign scheduling parameters in such a manner so as to minimize the stack memory usage of the system. Random generated mixed-critical runnable sets have been used for the performance evaluation, which later demonstrated that the presented approach can drastically reduce the memory stack usage of the system. Targeting the same systems that comply with the AUTOSAR standard, Nesredin Mahmud et al. [24] aim to optimize the allocation of fault tolerant embedded software applications. The presented optimization is based on an Integer Linear Programming model that minimizes the total power consumption of the system while taking into consideration the imposed timing and reliability requirements. The evaluation on synthetic automotive applications had shown that the proposed optimization approach effectively applies to small and medium-sized fault-tolerant distributed applications but is not scalable for complex applications.

Trying to enable an offline analysis of an in-vehicle fault diagnostics system, Shilpa Raju et al. [25] propose a mechanism for establishing a temporal relationship of the logged fault data across the vehicle. As the vehicular network consists of heterogeneous ECUs, each having its own local time, a correlation of fault data can lead to inconsistent results. The authors propose a synchronized global time base that provides a solution for a common time stamp across the vehicular network. Tests conducted on an actual hardware setup have resulted in a successful time synchronization between the time slaves and time master.

### 3.2. AUTOSAR Testing

The probability of software faults that can lead to severe consequences has increased with the large number of software components installed in modern vehicles. These components consist of millions of lines of code that require adequate testing. In order to test these software components in the AUTOSAR architecture, hardware-in-the-loop simulations are used by the testers. However, these simulations have their own limitations as the software components cannot be tested at early stages. Addressing the functional safety aspect in the automotive industry, Jihyun Park and Byoungju Choi [26] highlight the importance of software fault injection testing based on AUTOSAR. The authors propose a method of fault injection and define types of software faults that might occur. These faults can arise as a result of the call relationship between the different AUTOSAR layers. The presented method is applied during the software development process and can inject various faults of the AUTOSAR based automotive software. Furthermore, the solution is implemented as ASFIT and compared to already existing fault injection methods. An empirical study was conducted by a Korean motor company and confirmed the superior performance of the method. Stating that tests of the real-time operating system module of AUTOSAR face different challenges, Kazuto Shigihara et al. [27] introduce a test program generator to solve the problems. One of the challenges of testing the AUTOSAR OS is the large amount of required test cases, thus leading to time consuming executions. Further challenges are clarified during their work, and a novel test program generator is described. Moreover, in order to solve the constraints on non-trusted operation system applications, the authors developed a test library. The effectiveness of the approach was proved by the conducted tests on commercial implementations of the AUTOSAR OS.

Focusing on the gap of testing techniques based on software-in-the-loop in AUTOSAR, Sooyong Jeong and Woo Jin Lee [28] present an automated testing technique. As there are no automated techniques of testing AUTOSAR components using SiL simulations due to the involvement of closed-loop controls and feedback, manual testing prevailed. In order to use the automated testing module that is presented as an integration to the existing SiL simulator, test cases including the closed loops are converted to a temporal form by using a test case conversion method. The efficiency and effectiveness of the proposed automated testing method is shown as a result of the conducted case study. Following a similar approach, Andrija Mihalj et al. [29] present an existing Advanced Driver-Assistance System environment testing system that creates a test environment for the communication simulation in the middle layer of the AUTOSAR architecture. The created generator and test environment are used to test the communication between the control units. The test environment generator is actually a Python based program that processes the ARXML. Furthermore, a qualitative check of the ADAS system’s performance can be done by using different test environment generator configurations. The proposed test environment generator consists of a parser, data storage, and a generator. However, the conducted tests highlighted the importance of future work that is required to accelerate the execution time and introduce stable methods for data storage.

### 3.3. AUTOSAR Remote Testing

The increasing connectivity of modern vehicles can be regarded as one of the most notable aspects of the automotive industry. At the same time, the development of communication technology offers a high potential of development of the Internet of Things. Merging the two opportunities together, the existence of AUTOSAR provides the opportunity to integrate existing Internet of Things technologies to modern vehicles. In their work, Marko Dragojević et al. [30] propose a solution for using IoT technologies in order to remotely diagnose modern vehicles. Their work uses already existing IoT technologies to enhance the automotive middleware, Adaptive AUTOSAR, in order to enable remote diagnosis and monitoring. The presented architecture demonstrates an easy integration of IoT into the Adaptive AUTOSAR, making use of a simplified functionality. The work is concluded by stating that the proposed solution has certain overheads. However, the purpose of their work was to give an indication of feasibility of the current IoT technology in the automotive industry. Aiming to update the software based on IoT solutions, Stevan Stević et al. [31] present their approach of integrating IoT technologies with the Adaptive AUTOSAR platform. The solution consists of a software architecture upgrade, a cloud connectivity based on IoT, and extensions of the Adaptive AUTOSAR stack. The chosen IoT technology is OBLO, which is used as a back-end part of the system, allowing an over-the-air update for modern vehicles. The new Adaptive AUTOSAR platform, together with OBLO, aims to solve problems regarding expensive and slow updates.

With more and more radar solutions introduced in automotive applications, standard criteria compliance needs to be proven and performance needs to be assured. In their paper, Carlos Junio Rocha et al. [32] present an automated approach to allow over-the-air testing and validation in the production units of various radar units. This approach not only covers the pass/fail conditions but also aims to determine the radar antenna array radiation diagrams. The results of the presented work illustrate the importance of conducting the tests in a clean and shielded anechoic environment. Some of the unique challenges encountered in the automotive test environments are presented by Patrick Pelland et al. [33]. The authors state that the conventional over-the-air techniques are not adequate for testing complex systems such as modern vehicles. The concluding remarks of their work suggest that the next generation of automotive antenna measurement systems will be used for both over-the-air testing and antenna pattern measurements. These future amenities are to be equipped with positioning systems appropriate for vehicles with OTA test equipment in order to aid communication with the relevant wireless systems. Philipp Berlt et al. [34] highlight the importance of developing new test procedures as the available ones cannot be performed if the RF port is not accessible. The importance is also underlined by the relation between the wireless information and the vehicle state of operation.

Sreehari Buddappagari et al. [35] present in their paper a complete test system based on an over-the-air method for installed-performance evaluation of automotive radar systems. As radar systems are one of the most important aspects of autonomous and connected driving technologies, their functional performance must be thoroughly validated. The highest degree of realism is provided by real-world traffic scenarios. However, this method consumes a lot of resources and has many associated risks. The work makes use of an exemplary traffic scenario in order to validate the performance of the whole implemented system. The conducted tests show promising results, having similar simulated outcomes as the desired ones. Similarly, Sreehari Buddappagari Jayapal Gowdu et al. [36] regard the 77GHz radars as key drivers for autonomous driving and further highlight the resource consumption of traditional tests that must cover millions of kilometers with real test drivers. The presented approach aims to augment real field tests by using hardware-in-the-loop to emulate virtual radar environments. This solution allows simulation of the response behavior of various interconnected subsystems of a vehicle with having the radar-under-test installed on the vehicle. While the over-the-air method allows operating the radar sensor without having to modify it, the virtual environment aids testing for the correct operation of the same radar sensor. The authors have managed to create a holistic, reproducible, reliable, and scalable end-to-end testing concept. The article written by Michael Gadringer et al. [37] presents the development process of a simulator responsible for generating virtual radar targets in order to test radar sensors. As stated in the previous articles [35,36], the authors underline the risks of the widely used road tests and their associated risks and resource consumption. A Radar Target Simulator has been developed together with software responsible for generating the input parameters for the simulator. The testbed is used to conduct vehicle-in-the-loop tests in order to simulate the testing kilometers on road. The performance of the Radar Target Simulator was validated both in the laboratory and on a roller test bench. Moreover, the authors provide an overview of the radar sensor potential future development, stating that the trends will have a huge impact on the presented Radar Target Simulator system.

On the other hand, Nils Hirsenkorn et al. [38] address the importance of the simulation accuracy and the realism of the virtual sensors. Their paper describes a simulation approach for an automotive radar sensor beginning with the wave transmission, up to the intermediate frequency signal. Antenna power patterns, reflection, and ray divergence were taken into consideration in order to obtain a high fidelity. At the end, the model is deployed in Virtual Test Drive, a detailed driving simulator demonstrating comparable results. Aiming to obtain a low clutter environment for over-the-air radar sensor testing, and as a result of an initial analysis, Muhammad Ehtisham Asghar et al. [39] proposed an optimal absorber configuration. In order to detect clutter and unwanted scatters in the testing facility, the authors made use of a programmable radarbook and a reference radar sensor that has a well-known performance. The result of the work demonstrates the efficiency of the proposed method by an excellent detection of unwanted reflections.

### 3.4. Radar Optimization

With radar sensors playing an important role in a safe driving experience, the rapidly evolving automotive industry makes use of these sensors in order to improve the detection capabilities of moving objects. However, high resolution radar sensors and LiDAR lead to an extended target tracking problem. Karl Granstrom et al. [40] underline the problem faced when there are multiple detections per tracked object. Their paper presents a stochastic optimization method that solved the problem in a single step and maximizes the desired likelihood function. The sampling-based method was confirmed during an experiment with Velodyne data showing that the proposed filter is superior to any other previous method. Moreover, instead of relying on multiple algorithms, the solution outputs the desired likelihood directly. Also focusing on optimizing the classification of moving objects, Ali Walid Daher et al. [41] present a solution by applying machine learning. By using a Raspberry Pi for the training and testing purpose, IoT is introduced in their work. Rulex, a high-performance machine learning package, has been ported to the board in a client/server setup. The experiment has proven an accuracy of 100% for humans and 96.67% for vehicles. Aiming to improve the imaging of the moving targets and enhance the angular resolution, Shahzad Gishkori et al. [42] extend the forward-canning synthetic aperture radar methodology with a forward-looking automotive radar sensor. In order to distinguish between moving objects and stationary ones, the authors have adapted a nonparametric matrix decomposition to the forward-scanning synthetic aperture radar. During the work, optimization problems have been solved with a proposed alternating direction method of multiplier iterative methods. In the end, the validity of the proposed solution is proven by both simulation and real data.

## 4. Method

The system presented in this paper offers an optimization solution for the AUTOSAR communication implemented on a radar sensor. The sensor can be remotely accessed via an Arduino interface that was implemented from scratch. The research steps of our approach are implemented as follows:

1. Analyzing the literature review in order to understand the current approaches in AUTOSAR optimization and remote radar sensors (Section 3);
2. Building the system architecture that takes into account communication optimization, remote testing, and radar application testing (Section 4);
3. Implementing the Private Data Adapter (PDA) optimization solution (Section 4.1);
4. Constructing the radar sensor remote testing prototype (Section 4.2);
5. Implementing performance tests by constructing a Radar Production Mode (RPM) module (Section 4.3);
6. Analysing the conducted performance tests (Section 5.1 and Section 5.2);
7. Identification and discussion of the advantages and disadvantages of using the proposed optimization solution (Section 5.3).

**Figure 3:**
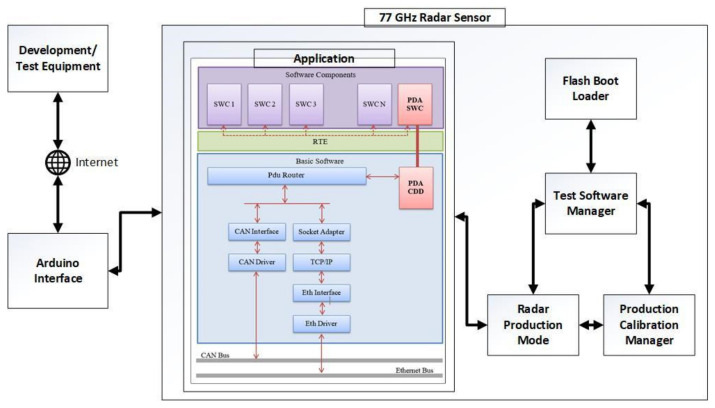
System architecture.

The system architecture is depicted in Figure 3 and is divided into three main parts. The application is the software component that runs on the sensor when it is mounted on the machine. This module represents the implementation of a communication software module, called a Private Data Adapter (PDA), which is used in a private communication system (sensor-to-sensor communication) and integrated in the AUTOSAR Communication.

The system is designed to meet the requirements of remote-testing (as proposed in RQ2), meaning that a developer working in specific conditions, outside of the normal in-site laboratory, will be able to access the radar sensor’s applications. The developer or tester connects via the internet to an Arduino interface while the radar sensor is connected to the interface defined inside the company’s laboratory. With the development of the fifth generation of radar sensors, there was a need for software that can test if the sensors are correctly assembled and to prepare them for commercial use. Radar Production Mode (RPM) is a software component that performs various tests and processes on the radar sensor and its application, such as memory usage and runtime tests. These tests are based on commands received on the CAN bus. Thus, RPM is a component working on a demand-response principle, with the only activities being done automatically are the initializations of the various drivers it needs in order to fulfill its purpose. This module is responsible for reading various voltages, temperatures, signals, and sections of memory. Moreover, another service offered by RPM is the sending of fixed structure messages on the communication channels available on the project on which it is located. These can be either CAN or Ethernet buses. The structures of the sent messages are to be checked by their receiver in order to confirm if the sensor communication works as expected.

The current generation of the RPM is loaded onto the ROM memory of the sensor, along with other software components. The upload is done by a direct flashing on the microcontroller’s memory. In general, each software component, at initialization, configures the hardware components of the sensor, but as this step was removed from RPM, it is based on the configurations made by the component that is run before it, Flash Bootloader (FBL). This limitation implies that any changes made to the configurations in the FBL can negatively affect the functionality of the RPM.

The Radar Production Mode (RPM) communicates with the Flash Bootloader, this being the first component to run on the sensor when it is turned on. This component deals with checking the integrity, in the sensor memory, of the other software components. This check is done by calculating the Cyclic Redundancy Check (CRC) of each component and comparing the result with the verification value that was attached to the component when loading into memory. Additionally, the FBL analyzes which of the software components are present at the sensor starting moment and determines, based on a priority, which component, verified and without errors, will start. There are two components that can be started by it, the sensor application and the Test Software Manager, which has priority over the application.

**Figure 4:**
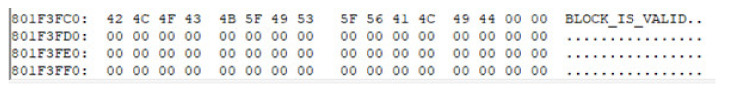
Validity model in RPM.

The Test Software Manager (TSM) works similarly to the RPM, based on the demand-response principle. This component waits for a request, in the form of a message sent on the CAN bus, and after processing the request, it determines which test component will check and start it. In the case of RPM, it will compare the validity model found in RPM with the one contained in TSM. The validity model is a predetermined sequence of values (Figure 4) that is written in the sensor memory and in the memory section of the RPM and TSM, respectively. If the validity models are identical, the TSM will start the RPM boot process, which consists of copying it from ROM to RAM and booting it from RAM. The deletion of the TSM from the sensor memory is performed by the RPM at the end of the testing process.

Production Calibration (PC) is the other component that can be started by TSM. It deals with the calibration of the radar antennas of the sensor, and its deletion from the sensor’s memory is also performed by RPM.

### 4.1. Private Data Adapter Module

The communication system of the 77 Ghz radar sensor is divided by a specific type of communication. It is formed by the specific vehicle communication between the ECUs and the sensors. In addition, a private communication is established between every two radar sensors. The OEMs’ requirements describe the type of communication the radar sensors are using. This can take place on CAN bus, Ethernet, or a combination of these two. Given the fact that a vehicle can be equipped with up to 16 radar sensors, eight pairs of such sensors can initiate communication on the private channels of the vehicle. Data as raw targets are exchanged on private communication channels between two radar sensors at a given time. This data is collected from the environment, and the result of the private communication will be the creation and transmission of objects and warnings towards the vehicle communication channels. The numbering of the sensors in a car starts with the front of the car. In this regard, the sensor identified as S0 will communicate on a private channel with sensor S1; sensor S2 will communicate with sensor S3, and so on. Given the fact that only two sensors can communicate on a private channel at a certain time t0, in order to simplify the software, a left-positioned sensor (named with an even identifier—S0-S14) will send messages with names ending in “_S0”. Meanwhile, a right-positioned sensor (named with an odd identifier—S1-S15) will send messages named with “_S1” at the end. Taking into consideration the large amount of data that will be transmitted on the private channels, the communication can be initiated on Ethernet or CAN FD. The configured arbitration rate of CAN FD is 1Mb/s, while the configured data rate is 2Mb/s. In addition, the Ethernet bus speed is configured to 100 Mb/s.

**Figure 5:**
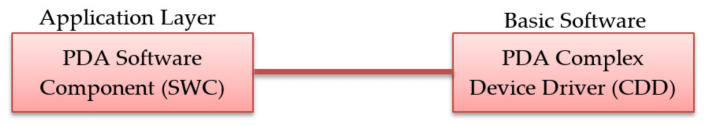
Private Data Adapter module design.

Our solution is called Private Data Adapter (PDA) and will be part of the 77 Ghz Radar Sensor software, at the level of AUTOSAR implementation. It is divided into two sections, namely a software component (SWC) and an AUTOSAR Complex Device Drive (CDD), as presented in Figure 5. These two components facilitate the communication on private channels. The implementation facilitates the messages’ forwarding in a 1:n manner, but at the same time also stores large amounts of data until these are processed. The PDA SWC is responsible for exchanging data between other SWCs and the Pdu Router. The PDA CDD represents an AUTOSAR Upper Layer in the Communication Stack and implements all the private messages that are transmitted or received by the PDA SWC.

**Figure 6:**
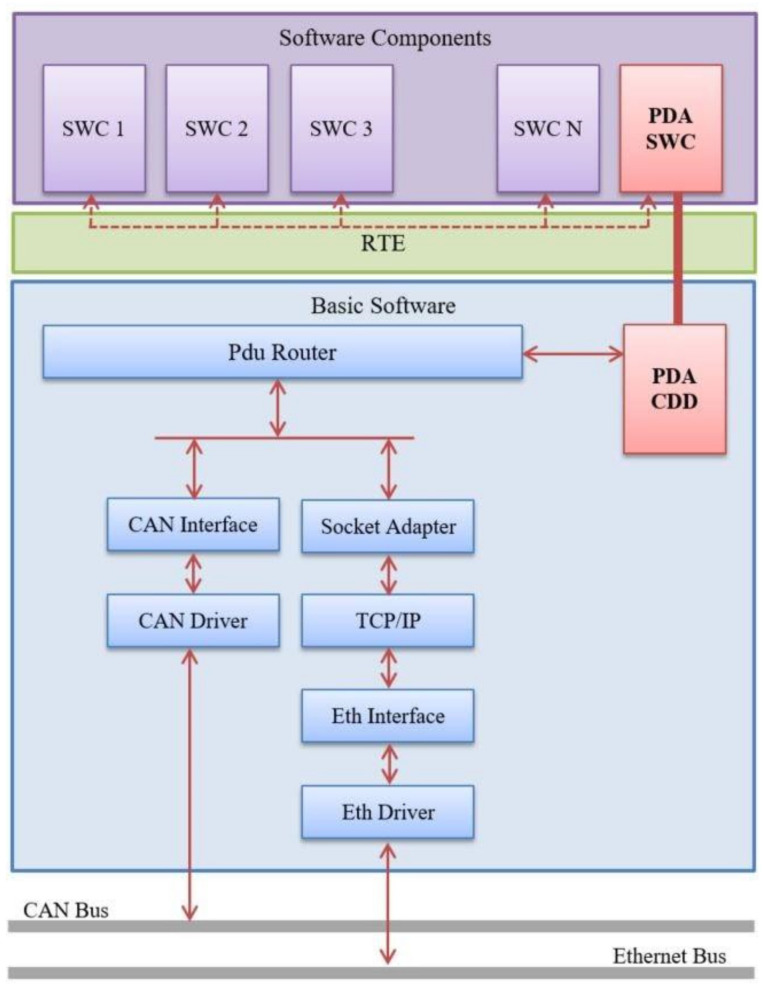
PDA module in AUTOSAR development process.

The PDA (Figure 6) uses dedicated software buffers for transmission/reception of each message and runs cyclic at every 60 milliseconds. Meanwhile, it performs both transmission and reception of messages and communicates with other SWCs (e.g., algorithm modules) via RTE and directly with the Pdu Router.

The structure of PDA SWC is realized based on several submodules, such as the transmission of each message, the reception of each message, and PDA specific functions. Besides the transmission and reception of messages, the PDA module is responsible for the following actions:

- Signals computation, which is meant to apply a factor and/or an offset to signals in order to transmit raw values on CAN/Ethernet and to remove it at reception in order to obtain the physical values needed by other SWCs;
- Range check, according to the range defined in DBC and default values assignment (min value/max value) for signals out of range;
- End-to-End (E2E) protection for messages that are received, being accomplished as a CRC signal to verify data integrity and a counter signal that checks for lost messages.

In order to avoid a CAN FD bus conflict, the AUTOSAR Communication Stack uses post-build selectable variants defined for each sensor identifier. On all even variants (S0, S2, and so on) the messages are named with an ending of “_S0”. On all other variants, the messages’ names ends in “_S1”. Only two sensors are connected on a CAN bus, therefore only two different identifiers are required for each message. PDA is responsible for reading the sensor ID and to transmit the message to PduR with the corresponding Pdu Handle ID. As Ethernet does not depend on the message ID, only “_S0” messages are defined in the Communication Stack, and PDA sends it without depending on the sensor identifier. For example, for Sensor ID = S0, the CAN frame message will be <MessageName>_S0, while for Sensor ID = S1, the CAN frame message will be <MessageName>_S1, etc. For the Ethernet Frame, the format will be the same for all the IDs, namely <MessageName>_S0.

**Figure 7:**
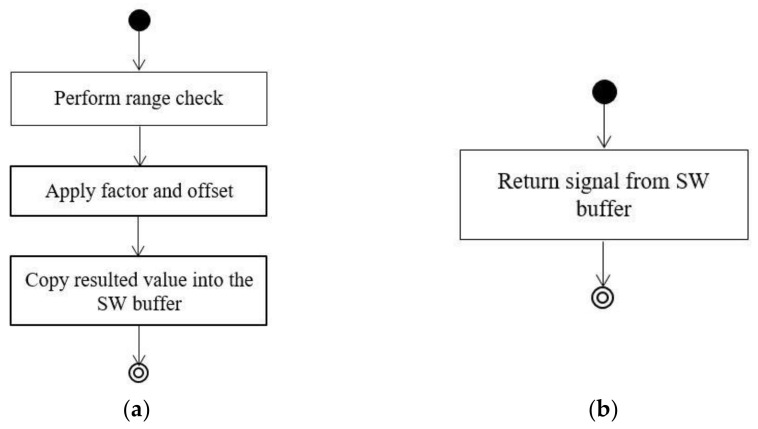
(**a**) Setting the signal to software buffer. (**b**) Getting the signal from software buffer.

The PDA module implements two types of messages: simple messages (up to 64 bytes) and complex messages of type Header-Data-Trailer, where multiple Data messages can be transmitted/received in a cycle. The second type of messages are defined when a message size exceeds the 64 bytes that can be defined on CAN FD. The last three bytes in a message that is both sent and received are dedicated to the end-to-end (E2E) protection: a cyclic redundancy check (CRC) signal and a counter signal. The complex messages of type Header-Data-Trailer allows data messages to be transmitted multiple times in a cycle. For each signal defined, a set and get function is implemented in order to write/read the signal to/from the software buffer and also to compute the signal. For example, if a certain signal *One_u8* of *MessageTwoData* message has a factor of 0.01, an offset of −20, and a range, [−20.5,20.5] are defined. The operations of setting the signals to a software buffer, as well as obtaining these from a software buffer are presented in Figure 7a,b.

#### 4.1.1. PDA Transmission

Each message defined in the DBC (CAN FD) or arxml (Ethernet) file has a corresponding sending signal submodule. Usually a message is implemented to be transmitted on both channels in a 1:n approach. However, there are cases when it is only implemented and transmitted on a single channel. 

**Figure 8:**
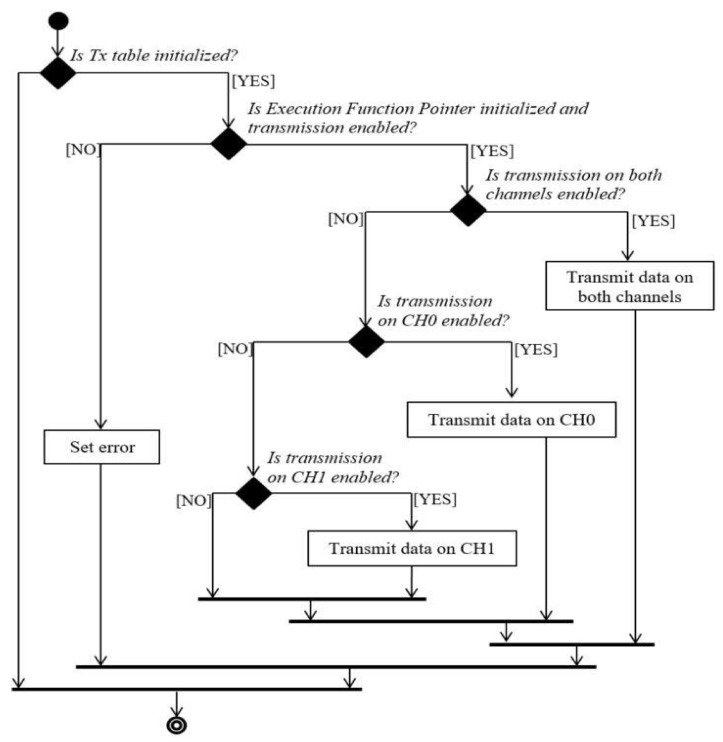
Transmission configuration diagram.

The transmission of all messages is done in a cyclic manner, at every 60 ms. If the transmission is initialized and the transmission is enabled, the data will be transmitted on the channels. Otherwise, the data will be transmitted on CH0 or CH1, depending on their availability. The process is described in Figure 8.

**Figure 9:**
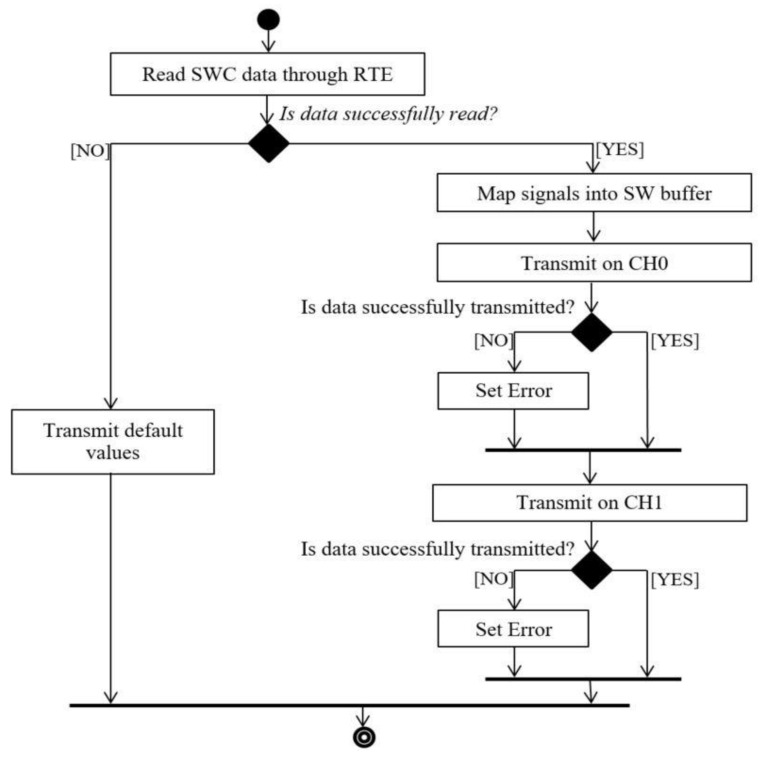
Message transmission diagram.

The message transmission steps are presented in Figure 9. Here, the data that should be transmitted is read from SWC through an RTE interface and is mapped afterwards into the PDA software buffer signal by signal. Apart from the signals read from the SWC, the software buffer may contain (only if the message will be received by the other sensor) two more signals dedicated to the end to end protection. The software buffer information is copied into a Pdu Router data type and the transmission to the Communication Stack is done by calling the PduR_PdaTransmit function with the corresponding Pdu Handle ID.

The transmission of a Header-Data-Trailer message type contains three different functions, one for each message, which is the reason why the PduR_PdaTransmit will be called multiple times in order to transmit multiple Data messages.

#### 4.1.2. PDA Reception

**Figure 10:**
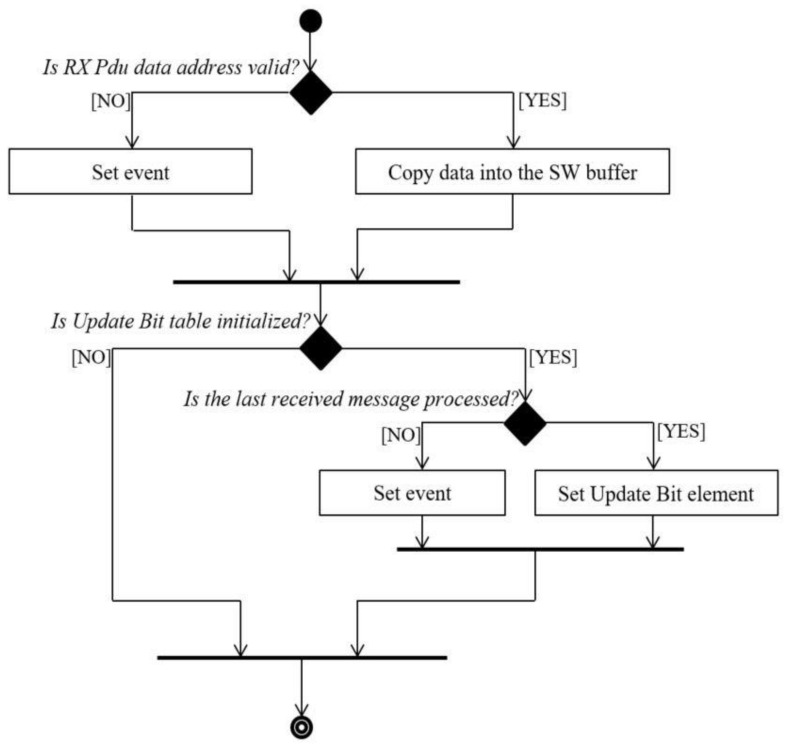
PDA reception through the Pda RxIndication function diagram.

The reception of messages is implemented in two steps. The first step assumes to copy the data received from the bus into the PDA software buffer when the Pda_RxIndication callback function is called (reception mode is implemented in Communication Stack as interrupt). In this function, an update bit is set to “TRUE” in order to indicate that a new message was received (Figure 10). In addition, each message that is received has a corresponding update bit. If more messages are received and no message was processed since the last processed one, the update bit will be set only once and the buffer will contain the newest data available for processing.

The reception does not depend on the sensor identifier as the AUTOSAR Communication Stack uses the same Pdu Handle ID for a message in all its variants. The PDA reception tables are aligned with the Pdu Handle IDs, and it is also not dependent on the sensor identifier. If MessageOne_S0 and MessageOne_S1 messages have a Pdu Handle ID of “0” defined in the Pdu Router, then Pda_RxIndication() call will be set on reception. In this case, if the data is available, it will be copied into the buffer. If the update bit table is initialized, then the update bit is set.

**Figure 11:**
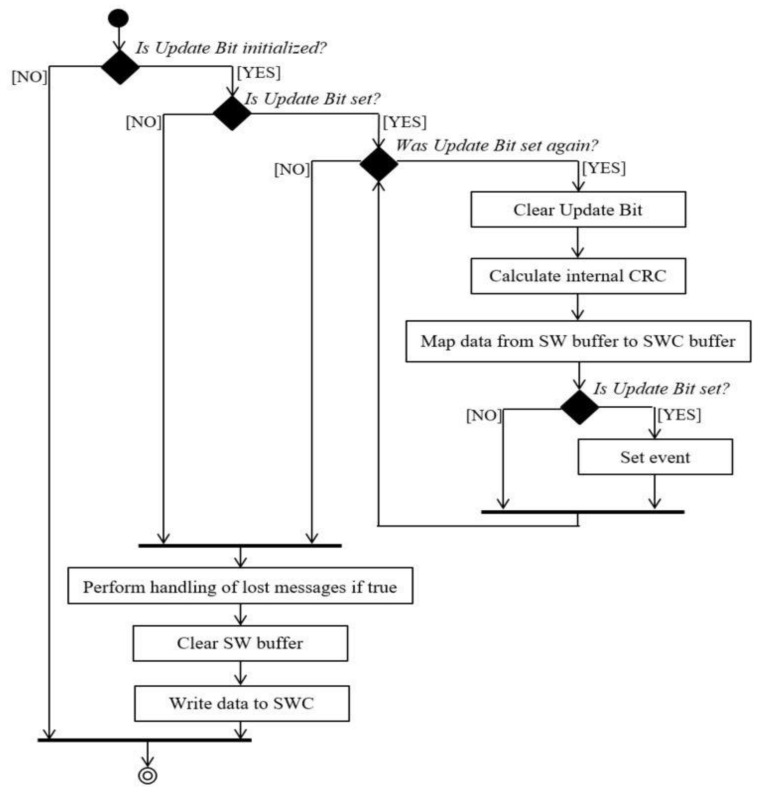
Processing the received data.

In the second step, data is processed, which means that each signal is unpacked and copied into an SWC data format type. The update bit is cleared (set to “FALSE”) first, before processing the data. Afterwards, the new data is transmitted to the SWC through RTE. This step is done cyclicy. In case the same message is received multiple times in a cycle, the data will be re-processed (for a defined amount of times), which always means that the “last is best”. The processing part of the reception is done cyclically every 60 milliseconds. The reception of a Header-Data-Trailer is similar to the reception presented above, except that, in this case, for each message (Header, Data, Trailer) an execution function is implemented. The update bit is set for each message in Pda_RxIndication (Figure 11).

### 4.2. Remote Access and Testing 

**Figure 12:**
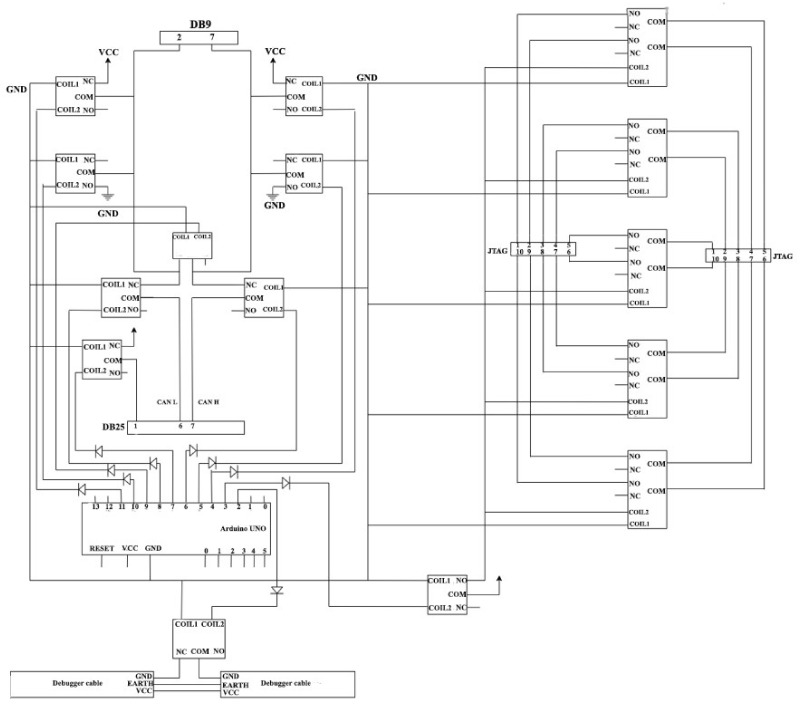
Radar sensor connection to Arduino.

The remote testing feature is formed due to the present necessities of accessing the radar sensor applications from a location outside the company’s physical boundaries. The sensor is connected to an Arduino interface, as presented in Figure 12.

The Arduino pins are connected to the relay control pin, JTAG connectors (used for debugger), DB25 (used for sensors), DE9 (used to connect to CAN), and debugger power cord. The Arduino digital pin 3 is connected to the relay control pin. The GND interrupted wire is connected to the NC (Normally Closed) pin, which means that the debugger will normally be powered. For safety purposes, a diode was used, being placed on the wire that connects the Arduino to the relay, to protect the board in case of a short circuit.

In order to implement a debugger ribbon functionality, the debugger is connected to the JTAG port via a 10-pin ribbon. This connection is needed in order to flash the newly implemented code on the sensors. In case of any arisen problems or bugs in the code, the radar sensor will reset. This behavior cannot be observed if the ribbon is connected, as the debugger does not allow the sensor to reset. Therefore, the testing must be conducted without having the ribbon connected.

The 10-pin ribbon requires 10 relays with a single contact to cut off the power. A solution consisting of five relays, each having two contacts, was chosen. The Arduino generates a current with a maximum voltage of 5V, which is too low to close all five relays at once. A sixth relay was used and connected to Arduino, being powered by an external source on Normally Open. With the help of this relay, the other five relays that are connected in parallel are controlled so that the voltage applied to each one is the same. The first relay will connect the ribbon from the debugger, with the 10 pins of the ribbon being still connected to the relays so that it is possible to interrupt the current. The pins of the second relay will be connected to the output of the relays and a ribbon that will make the connection to the sensor will be connected to it.

Through the DB25 connector, the sensor is supplied with current, more precisely through pin number 1. In order to reset the sensor, it is necessary to interrupt the power supply.

**Figure 13:**
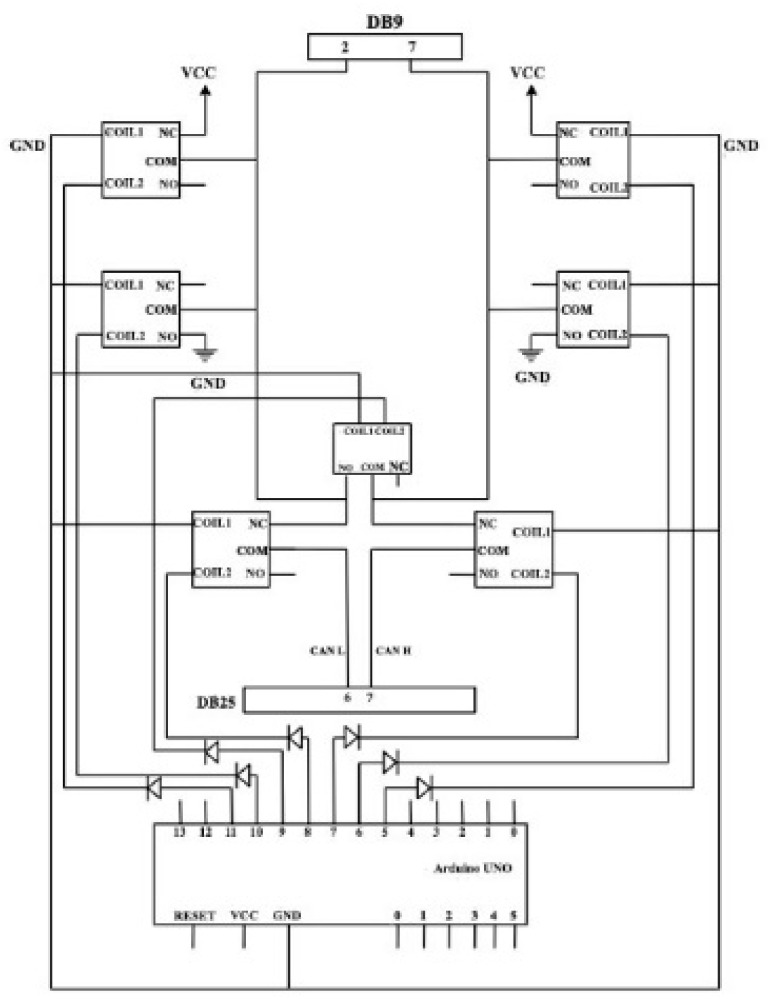
Buss-off and overload functionality.

**Figure 14:**
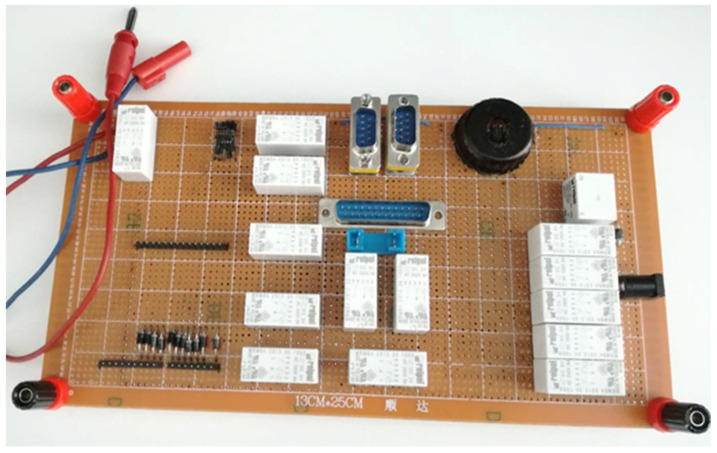
Radar interfacing module.

For implementing the bus-off functionality, three relays are needed, having the following connections: CAN H to GND; CAN L to VCC; and SHORT H to L. For the implementation of the overload (Figure 13), two relays, CAN H and CAN L, are needed. To determine how the sensors react in special situations, specific connections should be established, such as CAN H to VCC and CAN L to GND. The radar sensor interfacing module is depicted in Figure 14.

### 4.3. Tests on the Sensor’s Application

The Radar Production Mode (RPM) is responsible in our system for performing the necessary tests on the radar application in order to measure the performance results of the Private Data Adapter module. From the perspective of the RPM, these tests are treated as services.

The first time the RPM module is called, it calls the initializations of the other modules, except for the BSW module in AUTOSAR. In addition, it records all the tasks that are necessary to perform the services, after which it configures all the necessary pins and registers. After completing all of these steps successfully, it triggers a message on the CAN bus, signaling that RPM is ready to receive a request message.

**Figure 15:**
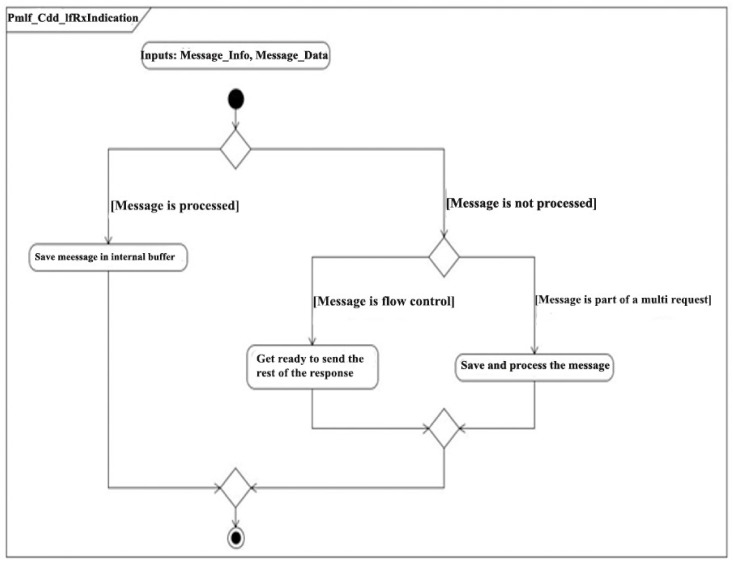
Rx Indication functioning.

There are two types of requests: single frame requests, which are simple requests whose data is contained in the 8 bytes of a CAN message, and multi-frame requests, which require several messages to be able to transmit all the required data. In the case of multi-frame requests, the first message is sent, followed by a message from RPM. This message is called Flow Control and indicates that the RPM is ready to receive the remaining messages. When a message is received on the CAN bus, the CAN module sends the message content to the module via a function called Rx Indication. This function determines, based on the status of the RPM, what type of message was received. It then processes it and saves its contents in an internal buffer (Figure 15).

**Figure 16:**
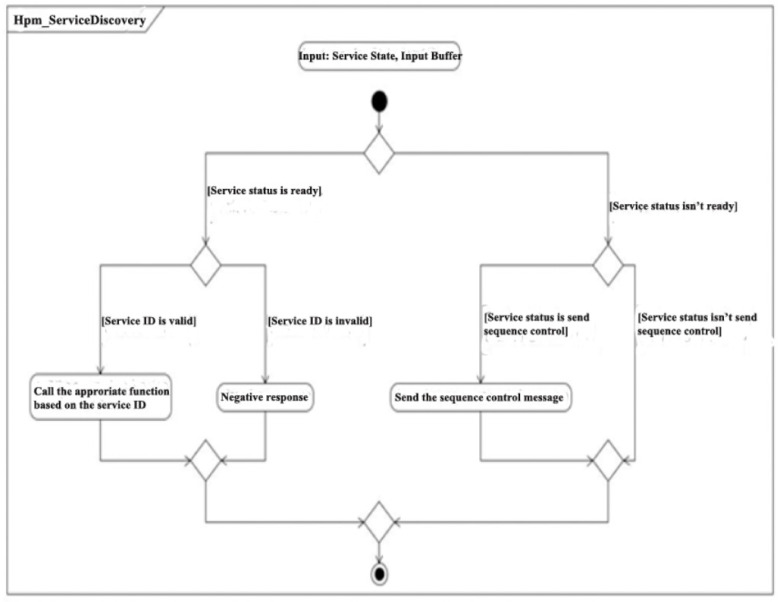
Service Discovery functioning.

One of the tasks that is called once every millisecond, is called Service Discovery. This parses the content of the message and checks if the structure and its data are valid. Next, it checks the status of the RPM. If the RPM is ready to perform services, it moves forward with execution. If additional data is required to perform the service, a message is triggered signaling this. If it is not yet ready to perform services, it waits until the status changes. Once the RPM is ready and moving on with the execution, it determined from which group the requested service belongs, and the contents of the message is sent to the filtering function of that service group. This step is contained in the activity diagram in Figure 16.

The filtering function of the group to which the requested service belongs takes over the content of the message, after which it checks if the requested service exists. If the service exists, the service is called and, where appropriate, the execution parameters extracted from the message are sent to the service. As an example, these execution parameters can be the value written by a writing service, or the time interval between messages and the number of messages to be stent by the test service of the chosen communication channel. Depending on their complexity and configurability, there are also services that have their own filtering functions.

If, during the processing of the request, it is determined that the request is invalid, either due to the structure of the content of the message or due to its incorrect values, the processing stops and indicates that the request is invalid by sending a negative message containing the error code relevant to the error encountered in the request message. Once called, the service tries to fulfill the request. In the case of success, it triggers the sending of a positive message that contains the result of the service. If it is unable to comply with the request, it triggers a negative message containing the error code relevant to the reason why it was unable to comply.

## 5. Results

This section implements steps 6 and 7 from the research methodology presented in Section 4. Firstly, the memory usage and runtime measurements are investigated by means of services in the Radar Production Mode. Furthermore, an analysis is performed on both PDA via Pdu Router and Full AUTOSAR solutions, and the advantages and disadvantages of both solutions are compared. 

### 5.1. Memory Usage

The testing setup used during the experiments was created in a partnership between the university and a local automotive company. The 77 Ghz radar sensor was connected to the Arduino interface in the company’s headquarters. Therefore, the software developers or testers had the possibility to connect to the Arduino interface remotely, via the internet.

The transmission of three different messages (of different types) was implemented based on the AUTOSAR approach and was compared with the transmission of the same messages implemented based on the Pda via Pdu Router approach:

- a simple message;
- a complex message (Header-Data-Trailer);
- a multiplexed message (that assumed the introduction of IpuM AUTOSAR module in AUTOSAR approach software).

The simple message is named MessageOne and defined with a length of 64 bytes. The complex message is named MessageTwo, and MessageTwoData can be sent up to 40 times in a cycle. The MessageTwoHeader message has a length of 24 bytes; MessageTwoData has a length of 64 bytes, and MessageTwoTrailer has a length of 3 bytes. The multiplexed message, MessageThree, has four different layouts and each one is distinguished and transmitted based on a selector field (multiplexer). Its length is 20 bytes.

All other messages are implemented in the PDA via Pdu Router module approach. The difference between the two approaches are located in the following files:

- Pda_Send_MessageOne_Exec.c;
- Pda_Send_MessageTwo_Exec.c;
- Pda_Send_MessageThree_Exec.c.

The RAM (EMEM) and ROM (Program Flash) memory usage was investigated, and runtime measurements were performed on both solutions. The RPM is responsible for these measurements by using dedicated services, as presented in Section 4.3.

The module usage results are presented in Table 1 for the optimization solution, namely PDA via Pdu Router, in comparison with the results obtained using the classic AUTOSAR solution where no optimization solutions are implemented. It can be noted that, in the case of PDA via Pdu Router, the memory usage of Pda_Send is lower than in the case of Full AUTOSAR. This result applies to both EMEM and Program Flash memory blocks.

The object memory usage results for PDA via Pdu Router are presented in comparison with the ones obtained for Full AUTOSAR and are to be found in Table 2. It can be noted that the highest improvement has been achieved in a complex Header-Data-Trailer message, while in the case of simple messages and multiplexed messages, the ROM memory usage has been improved by the PDA via Pdu Router optimization.

### 5.2. Runtime Measurements

The runtime measurements were performed three consecutive times (from start-up) for each message transmission using System Timer Register 0 (STM0) in a debugging environment as following:

- Starting point: For Pda via Pdu Router solution, the starting point was PduR_PdaTransmit(). The starting point for Full AUTOSAR solution was Rte_Write_<MessageName>().
- Ending Point: CanIf_TxConfirmation() in both cases.

It can be noted in Table 3 and Table 4, from the runtime measurements, that PDA via Pdu Router optimization offers an improvement at each tested message. Moreover, the most notable improvement is found with the multiplexed messages, in every single campaign that was performed. 

### 5.3. Discussion

**Figure 17:**
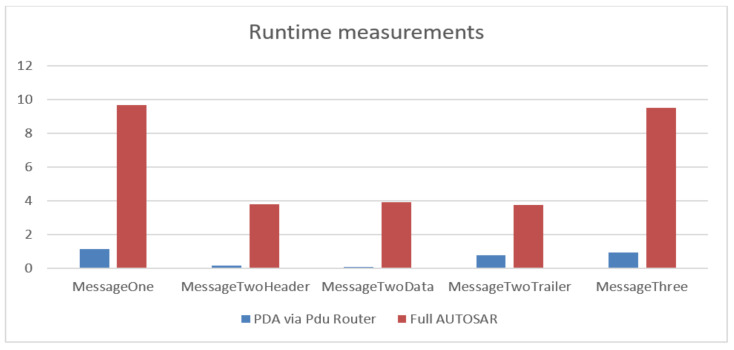
Runtime measurements comparison.

The (RQ3) of our paper is meant to analyze, in terms of performance, the gain of the proposed AUTOSAR communication optimization solution. Previous works on AUTOSAR radar sensor optimization have not addressed the communication issue at the presented AUTOSAR levels of our paper; therefore, from the literature review [3,5] we are referring to the evaluation methodology that aims to measure the performance. It can be noted from the previously presented experiments that the great advantage of PDA via Pdu Router solution is the execution time of the data transmission. It was proven to be considerably faster than the Full AUTOSAR solution. The results are concluded in Figure 17, where, for each message type (*x-axis*), the corresponding measured value is presented (*y-axis*).

**Figure 18:**
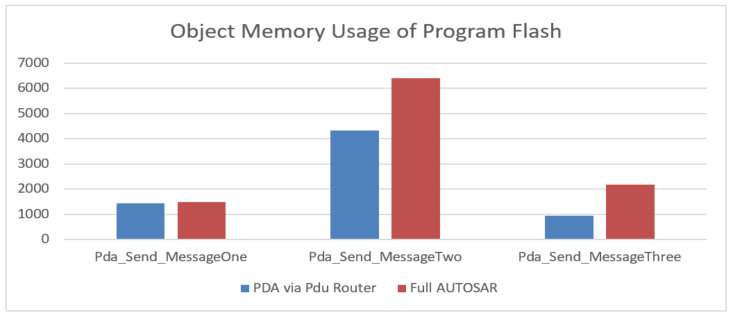
Comparisons on object memory usage of program flash.

In terms of ROM memory consumption, the PDA via Pdu Router optimization solution uses less memory resources compared to the Full AUTOSAR solution. The Full AUTOSAR solution uses almost two times the amount of memory resources. These results are presented in Figure 18 by highlighting the message type (*x-axis*) and the section length (*y-axis*). Another possible disadvantage of the classic AUTOSAR is its dependency on the DBC layout. With every change in the layout of a message (e.g., adding/removing a signal), the corresponding software buffer must be updated.

The PDA SWC abstraction represented by the interaction with other SWCs and the Com module through only the RTE highlights the major advantage of the Full AUTOSAR solution. Thus, this communication method allows independence from the PDU Handle IDs. Moreover, with the maintenance of the AUTOSAR Communication Stack code requiring less effort for the maintenance, another advantage of the Full AUTOSAR solution is underlined. In case a change is required in the Communication stack, based on the current DBC, the modules are updated through an automatically performed process for updating the configuration. When a new message is added in the DBC, the PDA via Pdu Router approach usage implies the deletion of the PDUs from the Com module and a new route to PDA CDD after the import. If the Full AUTOSAR approach is used, after the DBC import, no additional changes are needed. However, the RAM memory usage is lower during the usage of the PDA via Pdu Router approach.

## 6. Conclusions

This paper presents a solution for optimizing AUTOSAR communication as a response to our (RQ1). This approach assumes that the two connected radar sensors are always synchronized. In case of desynchronization (e.g., caused by an unexpected reset), the PDA behavior will be directly impacted by it. In order to address (RQ2), we have constructed an Arduino-based interface where the radar sensor is attached, allowing remote accessibility from any location. This interface offers the possibility to directly access the radar sensor’s application based on AUTOSAR, even during the current home-office Covid-19 imposed restrictions. For (RQ3), a separate software module was implemented, which is capable of conducting performance tests in order to determine what advantages our solution offers. Based on the results presented in the previous chapter, the PDA via Pdu Router solution presents more advantages in terms of memory usage and runtime measurements than the Full AUTOSAR. The PDA module is able to transmit and receive high amounts of data in a flexible way, as it is able to handle both event and time-triggered messages.

## Figures and Tables

**Table 1:** PDA via Pdu Router vs. Full AUTOSAR Module Usage.

		PDA via Pdu Router	Full AUTOSAR
Module	Memory Block	Section Length (Decimal)	
Pda_Config	EMEM	286	286
	Program Flash	1284	1284
Pda_Recv	EMEM	3397	3397
	Program Flash	2528	2528
Pda_Send	EMEM	3648	8901
	Program Flash	24,806	45,190

**Table 2:** PDA via Pdu Router vs. Full AUTOSAR Object Memory Usage.

		PDA via Pdu Router	Full AUTOSAR
ModuleObject Block	Memory Block	Section Length (Decimal)	
Pda_Send_MessageOne.o	EMEM	65	8
	Program Flash	1428	1472
Pda_Send_MessageTwo.o	EMEM	2525	7848
	Program Flash	4326	6398
Pda_Send_MessageThree.o	EMEM	25	12
	Program Flash	934	2170

**Table 3:** Runtime measurements results for PDA via Pdu Router.

PDA via Pdu Router			
Message	Starting Point [s]	Ending Point [s]	Runtime [ms]
MessageOne (simple)	2.96272992	2.96387129	1.14137
	3.01474416	3.01586722	1.12306
	3.06481028	3.06595220	1.14192
MessageTwo_Header (complex)	2.96127936	2.96143172	0.15236
	3.01119267	3.01134519	0.15252
	3.06128257	3.06143503	0.15264
MessageTwo_Data (complex)	3.96233074	2.96241716	0.08642
	3.01197675	3.01206331	0.08656
	3.06238609	3.06247233	0.08624
MessageTwo_Trailer (complex)	2.96360028	2.96360028	0.77455
	3.01233723	3.01311476	0.77753
	3.06242611	3.06320361	0.77750
MessageThree (multiplexed)	2.96182477	3.96277097	0.94620
	3.01257391	3.01349513	0.92122
	3.06266230	3.06359808	0.93578

**Table 4:** Runtime measurements results for Full AUTOSAR.

Full AUTOSAR			
Message	Starting Point [s]	Ending Point [s]	Runtime [ms]
MessageOne (simple)	2.96097376	2.97063171	9.65795
	3.01171761	3.02145308	9.73547
	3.06178232	3.07139313	9.61081
MessageTwo_Header (complex)	3.44568312	3.45036549	3.78237
	3.69668825	3.70039796	3.70971
	3.94922307	3.95179550	2.57243
MessageTwo_Data (complex)	3.44705112	3.45097265	3.92153
	3.69796526	3.70180876	3.84350
	3.94904268	3.95180012	3.75744
MessageTwo_Trailer (complex)	3.44692815	3.45065593	3.72778
	3.69702715	3.70066420	3.63705
	3.94813263	3.95065530	2.52267
MessageThree (multiplexed)	2.96163898	2.97119861	9.49963
	3.21158069	3.22111980	9.53911
	3.46162573	3.47108382	9.45809
